# Feasibility of HyperSight CBCT for adaptive radiation therapy: A phantom benchmark study of dose calculation accuracy and delivery verification on the Halcyon

**DOI:** 10.1002/acm2.70245

**Published:** 2025-09-09

**Authors:** Nicholas Nelson, Courtney Oare, Geoff Nelson, Thomas Martin, Jessica Huang, Hui Zhao

**Affiliations:** ^1^ Department of Radiation Oncology University of Utah Salt Lake City Utah USA

**Keywords:** adaptive therapy, cone‐beam computed tomography (CBCT), dose calculations, dosimetry, Halcyon, HyperSight, photon therapy

## Abstract

**Purpose:**

The development of on‐board cone‐beam computed tomography (CBCT) has led to improved target localization and evaluation of patient anatomical change throughout the course of radiation therapy. HyperSight, a newly developed on‐board CBCT platform by Varian, has been shown to improve image quality and HU fidelity relative to conventional CBCT. The purpose of this study is to benchmark the dose calculation accuracy of Varian's HyperSight cone‐beam computed tomography (CBCT) on the Halcyon platform relative to fan‐beam CT‐based dose calculations and to perform end‐to‐end testing of HyperSight CBCT‐only based treatment planning.

**Methods:**

A HU to mass density curve was measured for the HyperSight CBCT system and implemented into the Eclipse treatment planning system. Following this, computational dosimetric analysis was performed between dose distributions calculated on CT simulation (CT_sim_) and HyperSight CBCT images on two anthropomorphic phantoms for pelvic and head and neck treatment sites. Additionally, an end‐to‐end test was carried out for a head and neck intensity modulated radiation therapy (IMRT) plan.

**Results:**

The HU to mass density curves acquired on CT_sim_ and HyperSight CBCT were similar (< 30 HU) for near‐water equivalent materials, but deviated for high‐density materials, with a maximum difference of 150 HU. For dose calculations, excellent agreement between dose calculations performed on CT_sim_ and HyperSight CBCT phantom images was observed, where three‐dimensional gamma pass rates between the two dose distributions were observed to be ≥90% at 1%/1 mm (5% threshold). For the end‐to‐end test, absolute doses were verified to within 1% of ionization chamber measurements, while Delta4+ and portal dosimetry measurements yielded passing results (gamma pass rate ≥ 90%) down to 2%/2 mm criterion.

**Conclusion:**

In this study, the accuracy of dose calculations performed on HyperSight CBCT was found to be within 1% of CT_sim_ calculations for pelvic and head and neck treatment sites. End‐to‐end results using the RANDO anthropomorphic phantom indicate that HyperSight CBCT images are suitable for radiation treatment planning.

## 1 INTRODUCTION

There has been a growing interest in the development of adaptive radiotherapy (ART) in radiation oncology. ART allows for real‐time adjustments to radiation therapy plans based on patient anatomy changes or tumor response over the course of treatment using on‐board imaging methods, such as cone‐beam computed tomography (CBCT), ^1^, ^2^, ^3^ positron‐emission tomography (PET), ^4^, ^5^ and magnetic resonance imaging (MRI).^6^, ^7^ Although some imaging modalities have been implemented in ART, the focus of this study is on CBCT, which provides high‐quality volumetric x‐ray imaging at the treatment site and surrounding anatomy, enabling accurate daily assessments of patient setup and anatomical changes. ART can be implemented in both online and offline settings, allowing treatment modifications either immediately after CBCT imaging, prior to treatment (online), or in a subsequent treatment session (offline). Prior to the development of sophisticated CBCT solutions, limitations in terms of acquisition speeds and image quality, including spatial resolution and contrast, relative to traditional fan‐beam computed tomography (CT) on modern radiotherapy CT simulators (CT_sim_), have limited CBCT's utility in the realm of ART. Nevertheless, the development of on‐board CBCT marked a significant advancement in radiation therapy delivery, revolutionizing target localization through real‐time volumetric imaging of patient setup just prior to treatment.

The recent development of HyperSight technology (Varian Medical Systems, Palo Alto, CA) has led to significant improvements in acquisition time, image quality, and Hounsfield unit (HU) fidelity for on‐board CBCT imaging.^8^, ^9^, ^10^ HyperSight is currently available on the Halcyon and Ethos (Varian Medical Systems, Palo Alto, CA, USA) ring‐based gantry platforms and consists of a combination of improved detector hardware and reconstruction software that corrects for cone beam‐induced scatter and metal artifacts in an aim to provide fan‐beam quality CT imaging. It is hypothesized that this substantial improvement in image quality may allow for direct dose calculations and, thus, treatment planning directly on the acquired CBCT images, enabling online and offline CBCT‐based adaptive therapies.

The goal of this study is to provide a comprehensive overview of the HU to mass density calibration of HyperSight across multiple different scan techniques available and to evaluate CBCT‐based dose calculation accuracy relative to the conventional CT_sim_‐based methods using a CBCT‐specific HU to mass density calibration. Previous work by Bogowicz et al. evaluated the accuracy of dose calculations performed on HyperSight CBCT images relative to CT_sim_ using anthropomorphic phantoms.^11^ This study aims to provide another institutional experience and expand on such results through an investigation into HyperSight‐only planning and measurement‐based assessment of plan accuracy and deliverability (e.g, end‐to‐end testing).

## 2 METHODS

### 2.1 HU to density mapping of HyperSight CBCT

Accurate dose calculations performed on CT or CBCT datasets rely on the accurate mapping of HU values to mass or electron density values, depending upon the calculation algorithm being used to compute absorbed dose. The AAPM's Task Group 66 recommends that HU to density curves be measured individually for each scanner utilized for treatment planning at the time of commissioning and on an annual basis.^12^ The following section describes the methods used for measuring the HU to mass density curves on the Halcyon machine equipped with HyperSight CBCT to facilitate Acuros XB calculations.

**FIGURE 1:**
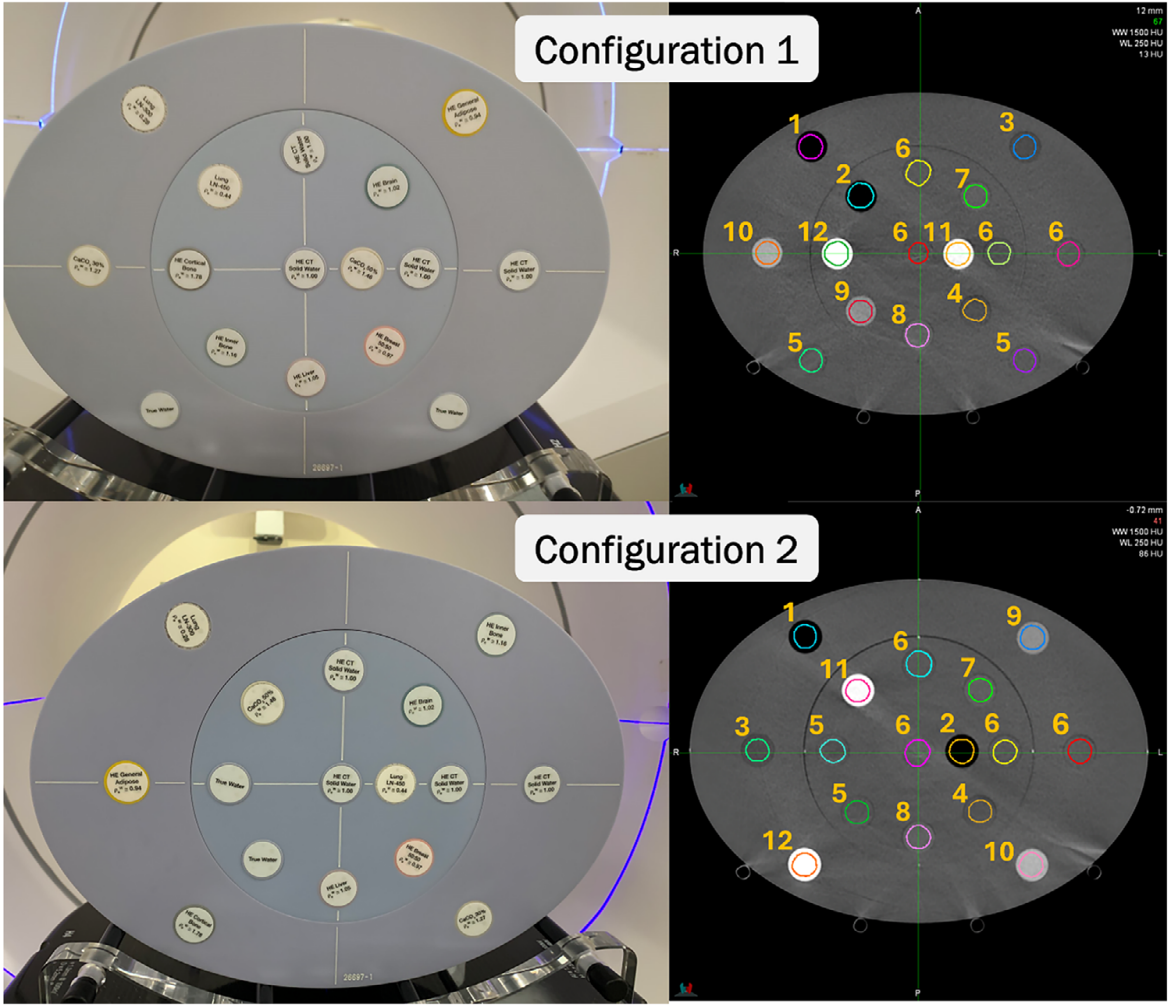
(Left) Photograph of the Sun Nuclear advanced electron/mass density phantom on the Halcyon couch top under two unique insert configurations (configuration 1 = top and configuration 2 = bottom). (Right) Axial slice of CBCT images (125 kVp, *iCBCT Acuros*) of the electron/mass density phantom for each respective insert configuration. The annotated numbers next to the inserts correspond to the insert numbers listed in Table 1. CBCT, cone‐beam computed tomography.

To perform dose calculations within the Acuros XB calculation algorithm, material specifications are accomplished through the relation of HU to mass density via a scanner‐specific calibration. From the derived mass density, Acuros XB will determine the material composition of each voxel in the image using a physical material table.^13^ The Sun Nuclear advanced electron/mass density phantom (Sun Nuclear, Melbourne, FL) was used to measure the relationship between HU and mass density on the Halcyon using the *iCBCT Acuros* reconstruction method and the following four scan options: 125 and 140 kVp, both with and without iterative metal artifact reduction (iMAR). CBCT imaging consisted of an in‐plane spatial resolution of 1 mm, a slice thickness of 2 mm, and exposures of 864 and 915 mAs for the 140 and 125 kVp scans, respectively. The density phantom supports the placement of sixteen known‐density inserts, and the HU–density curves were measured under two unique geometrical insert configurations, both of which are shown in Figure 1. For each image set, the regions of interest (ROIs) for each cylindrical insert were volumetrically contoured in MIM (MIM Software, Beachwood, OH, USA) using a circular brush with a diameter approximately 90% of the diameter. In terms of the contour length, the first and last two slices of the inserts were not included. Once contoured, the average HU within each ROI was averaged across the two geometrical configurations and correlated to the nominal mass density.

For comparison purposes, the measured HyperSight HU–mass density curve, derived from the 125 kVp *iCBCT Acuros* reconstruction method without iMAR, was then compared to clinically‐implemented HU‐mass density curves of two CT simulators: the Siemens SOMATOM go.Open Pro and Siemens SOMATOM Confidence. Both CT simulator curves were generated from 120 kVp scans, which is the kVp used for clinical treatment planning at our institution. The HyperSight CBCT HU–density curve was then imported into the Eclipse (Varian Medical Systems, Palo Alto, CA) treatment planning system (TPS, version 16.1) and was utilized throughout this study, effectively removing the effects of HU variation between imaging systems.

### 2.2 Treatment planning studies

To assess and benchmark the expected accuracy of dose calculations performed on HyperSight CBCT images, comparisons to identical calculations on CT simulation images were dosimetrically compared. Two phantoms were considered for this study: the BrainLab pelvis phantom (BrainLab, Munich, Germany) and the RANDO anthropomorphic phantom (Radiology Support Devices, Gardena, CA, USA). For the pelvis phantom, identical calculations were performed on the CT and CBCT images to evaluate calculation‐to‐calculation agreement. The Siemens SOMATOM go.Open Pro CT simulator was used in this study, and all dose calculations were performed using the Acuros XB photon dose algorithm in the Eclipse TPS using a 2 mm dose grid. Both CT_sim_ and CBCT image datasets used for planning and calculation slice thicknesses of 2 mm, while the CT_sim_ datasets had an in‐plane resolution of 1.17 mm, and the CBCT datasets had an in‐plane resolution of 1.05 mm.

As a further extension, intensity modulated radiation therapy (IMRT) plans were generated and optimized using the CT_sim_ and HyperSight CBCT images of the RANDO phantom. Although two separate CT_sim_‐ and CBCT‐optimized treatment plans were generated, calculation‐to‐calculation comparisons were performed by calculating the CT_sim_‐optimized plan on the CBCT image. The generation of two individual CT_sim_‐ and CBCT‐optimized plans allows for individual measurement‐based delivery assessment.

#### 2.2.1 Calculation comparison studies

In this portion of the work, the calculation of identical fields was performed on CT_sim_ and HyperSight CBCT images to evaluate the expected agreement between CBCT and CT_sim_‐based calculations under idealized conditions (e.g., no anatomical change). The fields evaluated consist of a 10 × 10 cm^2^ 6 MV flattening filter‐free (FFF) beam impinging upon the anterior surface of the pelvis phantom, a prostate stereotactic body radiation therapy (SBRT) plan delivered on the pelvis phantom, and a head and neck IMRT plan optimized for the RANDO phantoms. For the prostate SBRT plan, a clinical treatment plan previously treated at our institution was recalculated on CT_sim_ and CBCT images of the pelvis phantom, with the isocenter placed at a location representative of the prostate. The isocenters between the two imaging datasets were determined via rigid registration for both phantoms considered.

**FIGURE 2:**
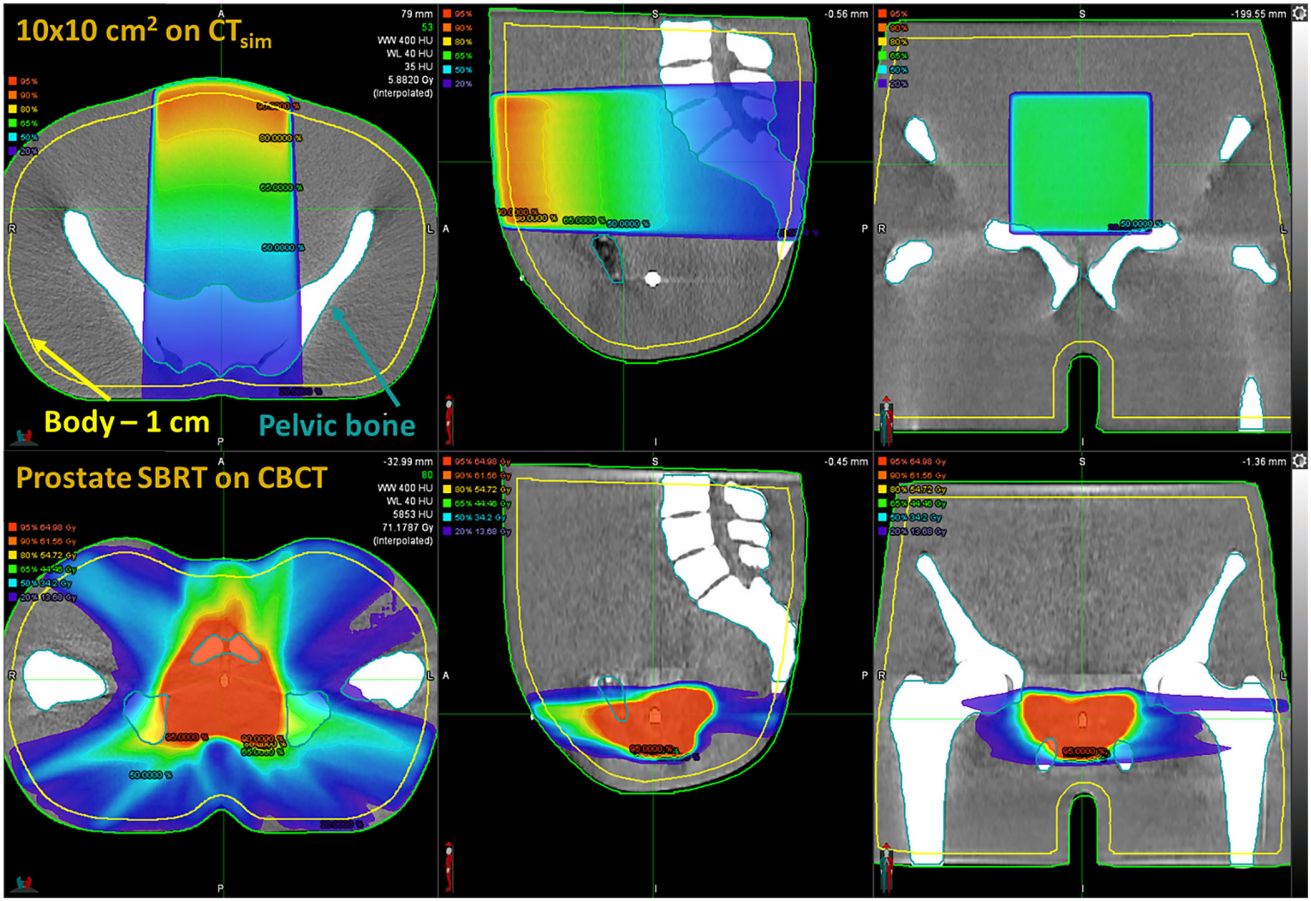
Illustration of dose distributions evaluated within the pelvis phantom for calculation comparisons. The top row displays a 6 MV FFF 10 × 10 cm^2^ field calculated incident on the CT image of the pelvis phantom. The bottom row displays a prostate SBRT treatment field that was calculated on the HyperSight CBCT image. CBCT, cone‐beam computed tomography; FFF, flattening filter‐free; SBRT, stereotactic body radiation therapy.

Comparisons between CT_sim_ and CBCT‐calculated dose distributions for all cases evaluated were performed on the CT_sim_ dataset using a rigid registration of the two images, where the CBCT dose was transferred to the CT_sim_ image for region‐based analysis of the two dose distributions. Specifically, differences (CBCT—CT_sim_) in the mean dose, dose to 98% of the volume (D_98%_), and dose to 2% of the volume (D_2%_) in the pelvic bone, the overall body minus a 1 cm retraction from the surface, and contours derived from the CT_sim_ isodose lines were evaluated. The mean dose, D_98%_, and D_2%_, were selected to gauge all points along the DVH curve to assess the high and low‐dose agreement. The 1 cm retraction from the surface/body contour was made to mitigate minor dosimetric differences that arise from minor registration errors and partial voxel effects of assessing and calculating dose near the surface. In addition to evaluating direct dose differences, three‐dimensional gamma analysis was also performed for a variety of criterion ranging from 3%/3 mm to 0.5%/0.5 mm. Gamma pass rates were computed using a low dose threshold of 5% to fully encompass a larger portion of the dosimetric space and evaluate agreement in high‐ and low‐dose regions. Figure 2 illustrates two of the fields evaluated in this portion of the study. It is important to note that the prostate SBRT plan was recalculated from a patient‐specific plan from our institution and may not reflect a clinical dose distribution in Figure 2.

#### 2.2.2 Clinical simulation of head and neck IMRT treatment

To further evaluate the dose calculation accuracy and the suitability of clinical treatment planning using HyperSight CBCT, an end‐to‐end simulation was performed using the RANDO anthropomorphic phantom. In addition to an end‐to‐end test of CBCT‐based treatment planning, the RANDO phantom was also used to facilitate further comparisons between CT_sim_‐ and CBCT‐calculated dose distributions. To accomplish this, the RANDO phantom was immobilized in an Alpha Cradle (Smithers Medical Products Inc., North Canton, OH) for reproducible positioning between CT_sim_ and CBCT, allowing for reproducible positioning on both imaging platforms and mitigation of geometrical changes in the RANDO assembly.

**FIGURE 3:**
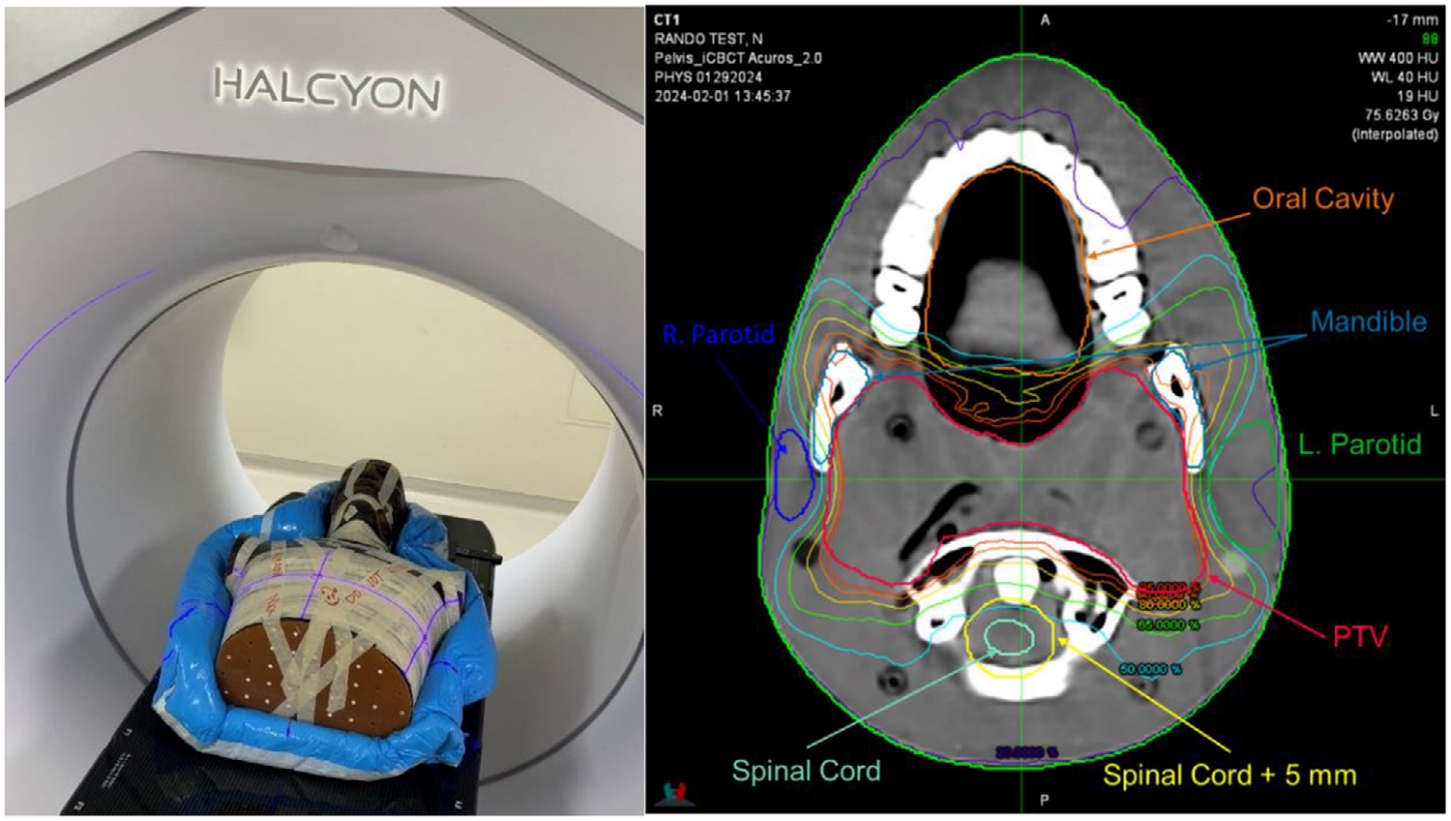
(Left) Photograph of RANDO anthropomorphic phantom immobilized within an Alpha Cradle on the Halcyon couch top for CBCT imaging. (Right) Axial slice of the CBCT image and optimized dose at the mid‐level of PTV (red) and relevant organs at risk (R. Parotid = blue, L. Parotid = green, Spinal Cord = light green, Spinal Cord + 5 mm = yellow, Mandible = light blue, Oral Cavity = orange). CBCT, cone‐beam computed tomography; PTV, planning target volume.

Following the simulation imaging on both platforms, a planning target volume (PTV) representative of a clinical head and neck target was contoured for planning, and organs at risk (OARs) were generated using auto‐contouring software (ProtégéAI, MIM Software Inc., Cleveland, OH, USA). The PTV contour was shared between the two image sets through a rigid registration and subsequent contour transfer, while the OAR auto‐contours were kept specific to the respective image sets. The NRG‐HN009 protocol was followed for treatment planning, which consists of a prescription dose of 69.96 Gy in 33 fractions to the PTV and OAR dose constraints placed on the parotid glands, submandibular glands, oral cavity, mandible, lips, spinal cord, cochlea, and brainstem. Both plans (CT_sim_ and CBCT) were optimized to meet dose constraints specified by the NRG‐HN009, and both yielded similar plan quality and multi‐leaf collimator (MLC) modulation. Figure 3 displays the RANDO phantom immobilized within the Halcyon bore for CBCT imaging and the CBCT‐optimized dose distribution with relevant contours. The difference in the amount of MLC modulation between the two plans was minimized through quantification of the maximum monitor unit duty cycle, which is the ratio of the total number of monitor units in a given plan to the fractional dose in cGy.

### 2.3 Delivery verification

**FIGURE 4:**
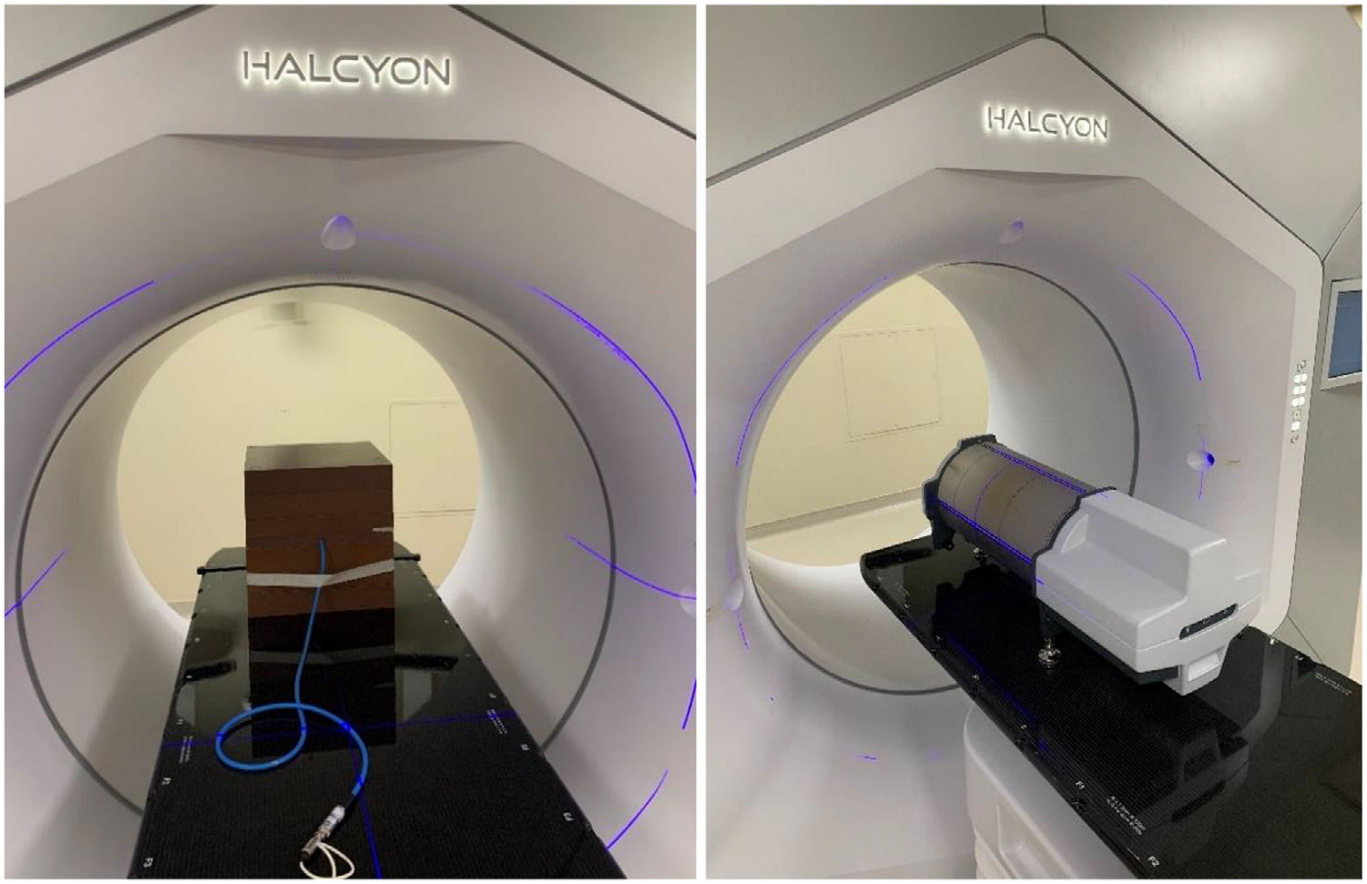
Photographs of (left) the ionization chamber measurement setup and (right) Delta4+ measurement setup.

The delivered dose of the CT_sim_‐ and CBCT‐optimized treatment plans was verified through in‐phantom ionization chamber measurements, portal dosimetry measurements using the on‐board electronic portal imaging device (EPID), and measurements with a Delta4+ phantom (ScandiDos, Uppsala, Sweden). For the ionization chamber measurements, the optimized plans were recalculated on a 30 × 30 × 30 cm^3^ stack of SolidWater (Model number 457, Gammex/RMI, Wisconsin, USA) and normalized to a dose of 280 cGy at isocenter. A Semiflex ionization chamber (PTW Dosimetry, Freiburg, Germany) was used to perform dose measurements at the isocenter within the SolidWater chamber insert. For EPID measurements, the portal dose image prediction (PDIP) algorithm provided in the Eclipse TPS was used to compute the predicted planar fluence map at the Halcyon's source to imaging distance of 154 cm. Delta4+ measurements were performed using longitudinal shifts to provide an effective spatial resolution of 2.5 mm in the central region of the detector array. Photographs of the experimental setups are shown in Figure 4.

## 3 RESULTS

### 3.1 HU to density mapping of HyperSight CBCT

Table 1 contains a tabulation of the derived HU as a function of mass density calibration for the four scan options evaluated. A maximum deviation in HU of 173 HU was observed for the highest‐density (cortical bone) insert across the four scan options, which was observed when switching from 125 to 140 kVp—an expected result due to the differences in the photoelectric effect for high‐*Z* materials. For materials with a density of 1.05 g/cc and below, the derived HU across all scan parameters evaluated was less than 20 HU. The influence of iMAR on the acquisitions had a very minimal impact on the derived HU for each of the material inserts evaluated, where maximum deviations of 5.8 and 3.6 HU for the HE Inner Bone insert at 125 and 140 kVp, respectively. Additionally, variations in the insert geometry led to the highest variability in the derived HU for the 125 kVp scans. These results indicate that for utmost dosimetric accuracy, a kVp‐specific HU to density curve should be employed for clinical use, or only one curve (and thus, kVp) should be used for clinical treatment planning and dose calculations. The work presented throughout this manuscript utilized the HU–density curve derived from the *125 kVp—iCBCT Acuros* CBCT scans.

**TABLE 1:** Summary of the derived HU values for the Sun Nuclear density phantom inserts for the four CBCT protocols evaluated across the two geometrical configurations considered (labeled as #1 and #2).

Insert information			*125 kVp—iCBCT Acuros*				*125 kVp—iCBCT Acuros with iMAR*				*140 kVp—iCBCT Acuros*				*140 kVp—iCBCT Acuros with iMAR*				
#	Insert Name	Density (g/cc)	#1 (HU)	#2 (HU)	Avg. (HU)	$\Delta_{1\to 2}$ (HU)	#1 (HU)	#2 (HU)	Avg. (HU)	$\Delta_{1\to 2}$ (HU)	#1 (HU)	#2 (HU)	Avg. (HU)	$\Delta_{1\to 2}$ (HU)	#1 (HU)	#2 (HU)	Avg. (HU)	$\Delta_{1\to 2}$ (HU)	$\Delta_{\mathrm{all}\;\mathrm{protocols}}$ (HU)
1	455 Lung LN‐300	0.29	−712.7	−694.8	−703.8	17.8	−711.8	−695.0	−703.4	16.8	−690.6	−681.8	−686.2	8.8	−688.5	−681.5	−685.0	7.0	18.8
2	485 Lung LN‐450	0.45	−538.6	−549.3	−544.0	10.7	−538.3	−546.7	−542.5	8.3	−531.2	−541.9	−536.5	10.7	−530.5	−536.8	−533.6	6.3	10.3
3	HE Gen Adipose	0.955	−53.9	−55.0	−54.4	1.1	−49.7	−54.8	−52.2	5.1	−55.6	−60.6	−58.1	4.9	−51.5	−58.1	−54.8	6.6	5.9
4	HE Breast 50:50	0.981	−34.1	−21.6	−27.8	12.6	−38.0	−25.2	−31.6	12.7	−37.2	−37.3	−37.3	0.1	−39.8	−36.8	−38.3	3.0	10.4
5	True Water	1	26.8	−10.4	8.2	37.1	29.0	−8.9	10.1	37.9	15.4	−23.2	−3.9	38.7	16.5	−20.4	−2.0	37.0	13.9
6	HE SolidWater	1.02	9.5	26.3	17.9	16.8	8.2	24.2	16.2	16.0	−5.6	13.5	3.9	19.1	−5.1	12.7	3.8	17.8	14.1
7	HE Brain	1.05	66.8	60.4	63.6	6.4	66.7	59.8	63.3	6.9	58.4	56.6	57.5	1.8	58.7	56.6	57.6	2.1	6.1
8	HE Liver	1.08	82.2	53.0	67.6	29.2	83.3	52.5	67.9	30.8	51.5	37.5	44.5	14.0	51.3	38.2	44.7	13.0	23.4
9	HE Inner Bone	1.211	333.1	334.1	333.6	1.0	339.8	339.0	339.4	0.8	287.9	302.6	295.2	14.7	291.5	306.1	298.8	14.6	44.1
10	CaCo, 30%	1.33	502.0	542.0	522.0	40.1	503.1	540.3	521.7	37.1	424.0	460.2	442.1	36.2	425.7	457.2	441.4	31.5	80.6
11	CaCo, 50%	1.567	1002.1	973.5	987.8	28.6	998.9	962.5	980.7	36.4	884.2	886.8	885.5	2.6	879.5	875.8	877.7	3.7	110.2
12	HE Cortical Bone	1.926	1541.5	1476.9	1509.2	64.6	1537.9	1479.1	1508.5	58.9	1342.1	1332.8	1337.5	9.3	1334.8	1337.6	1336.2	2.7	173.0

*Note*: In addition, the deviation in the derived HU across geometrical configurations are presented and the maximum observed deviation in mean HU across the scan protocols is evaluated.

Abbreviations: CBCT, cone‐beam computed tomography; HU, Hounsfield unit.

**FIGURE 5:**
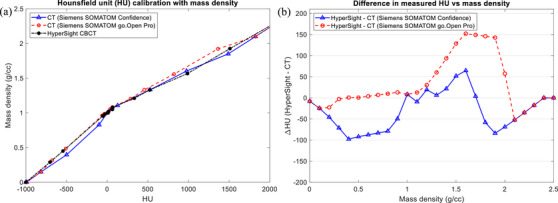
(a) Plot of the derived HU as a function of mass density for the Siemens SOMATOM Confidence CT simulator (blue, solid), the Siemens SOMATOM go.Open Pro CT simulator (red, dashed), and the Varian HyperSight CBCT (blue, solid). (b) The difference in derived HU as a function of mass density, defined as the difference between the HyperSight and CT_sim_ HU values. CBCT, cone‐beam computed tomography.

Figure 5 contains the HU‐mass density curves derived from the Sun Nuclear density phantom for the two CT simulators and HyperSight CBCT (*125 kVp—iCBCT Acuros*). In general, variations in the measured HU from CT_sim_ for the same mass density were minimal (< 100 HU) for mass densities below 1.5 g/cc, with a maximum observed deviation of up to roughly 150 HU at 1.57 g/cc for the Siemens SOMATOM go.Open Pro simulator, which corresponds to the 50% CaCo SNC insert or medium‐density bone.

### 3.2 Calculation comparisons (CT simulation and HyperSight)

**FIGURE 6:**
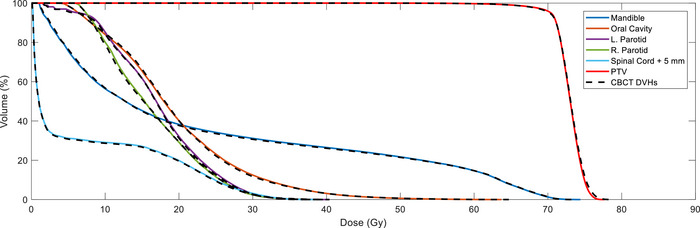
DVH data from CT_sim_‐optimized (colored solid lines) plan and DVH data from the CT_sim_‐optimized plan calculated on the HyperSight CBCT (dashed black lines) image for the RANDO head and neck IMRT treatment plan. CBCT, cone‐beam computed tomography; DVH, dose‐volume histogram; IMRT, intensity modulated radiation therapy.

Table 2 contains the results of the dosimetric analysis that was performed between fields calculated on CT_sim_ and HyperSight CBCT images of the pelvis and RANDO phantoms. Across these phantoms and the fields evaluated, differences in the calculated mean dose were within 0.5% across all contours evaluated, while the calculated *D*_98%_ and *D*_2%_ doses were within 3% across all geometries and contours evaluated. Figure 6 contains the dose‐volume histograms (DVHs) for the head and neck CT_sim_‐optimized plan and that same plan calculated on the CBCT image.

**TABLE 2:** Summary of dosimetric data derived across the two phantoms (pelvis and RANDO H&N) and three fields (10 × 10 cm^2^ and prostate SBRT on pelvis phantom, and head and neck IMRT fields on RANDO H&N) in terms of deviation in mean dose and dose to 98% and 2% of the respective volumes ($\Delta D_{mean}$, $\Delta D_{98\%}$, and $\Delta D_{2\%}$), expressed as a percentage of the prescription dose.

Phantom	Plan/Field	Contour (or isodose level)	Dose difference (CBCT—CT_sim_)		
			$\Delta D_{98\%}$ (%)	$\Delta D_{mean}$ (%)	$\Delta D_{2\%}$ (%)
Pelvis	10 × 10 cm^2^, 6 MV FFF	Body—1 cm	−0.4	0.0	0.3
		Pelvic bone	−0.4	0.0	0.3
		90% isodose	−0.7	−0.1	0.2
		50% isodose	−2.5	0.0	0.6
		20% isodose	−2.0	0.0	1.3
		5% isodose	−1.8	−0.2	1.8
		**Average**	−1.3	−0.1	0.8
	Prostate SBRT	Body—1 cm	−1.5	0.0	0.7
		Pelvic bone	−1.7	0.0	0.7
		90% isodose	−4.1	−1.3	0.3
		50% isodose	−3.6	−0.9	0.9
		20% isodose	−2.6	−0.4	1.3
		5% isodose	−2.3	−0.2	1.4
		**Average**	−2.6	−0.5	0.9
RANDO	Head & Neck IMRT	PTV	−2.8	0.2	2.3
		L. Parotid	−2.3	−0.1	2.6
		R. Parotid	−1.5	0.2	1.7
		Spinal Cord + 5 mm	−1.8	0.0	0.7
		Oral Cavity	−3.2	0.7	3.7
		Mandible	−2.1	0.5	3.0
		**Average**	−2.3	0.2	2.3

*Note*: Due to the limited number of anatomical structures on the pelvis phantom, isodose contours derived from the CT_sim_ dose distribution were generated to evaluate dose differences.

Abbreviations: CBCT, cone‐beam computed tomography; FFF, flattening filter‐free; IMRT, intensity modulated radiation therapy; SBRT, stereotactic body radiation therapy.

**FIGURE 7:**
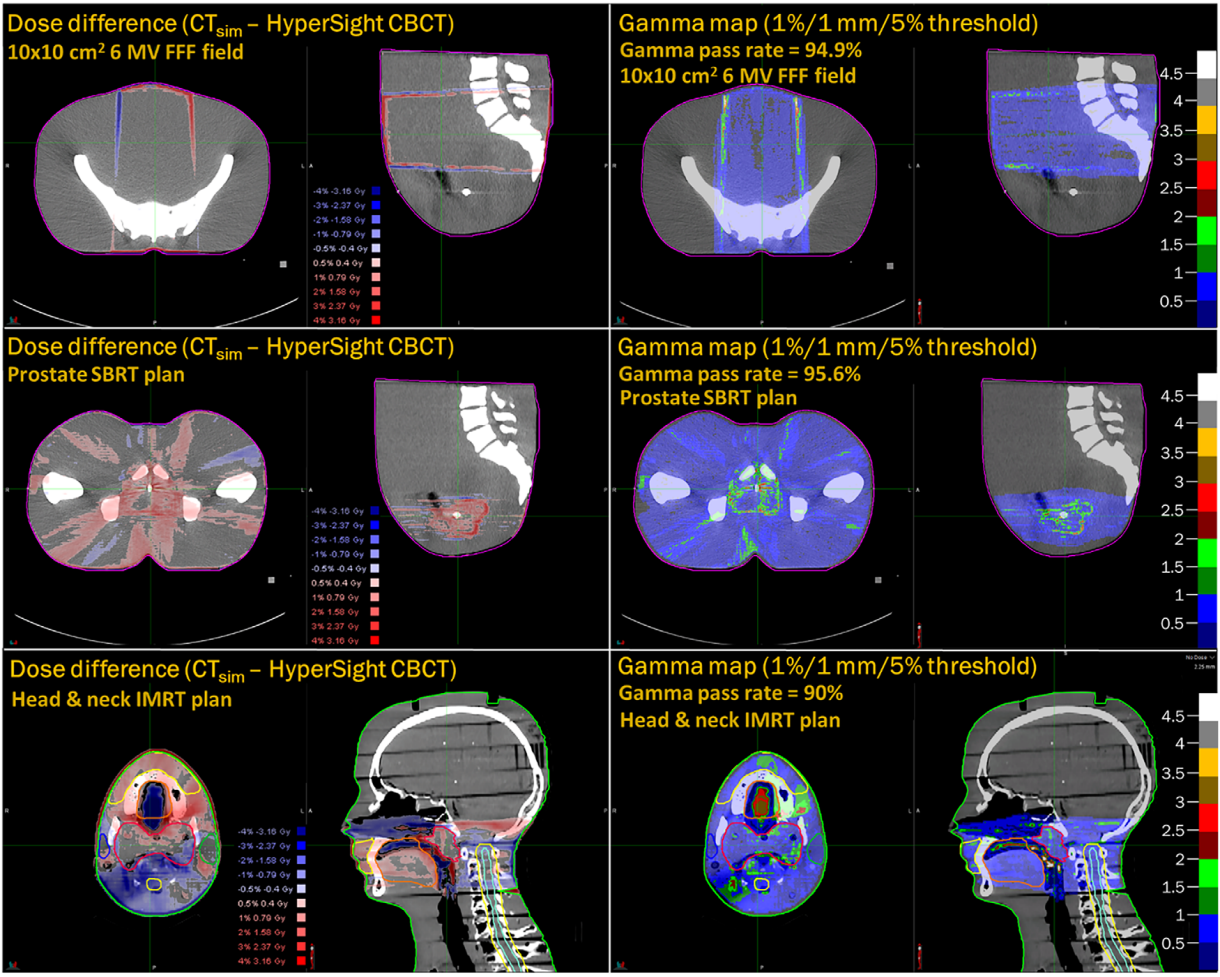
Axial and sagittal views of the dose difference (CBCT—CT_sim_, left column) and 1%/1 mm gamma function maps (right column) for the fields/plans evaluated in this work, consisting of 10 × 10 cm^2^ 6 MV FFF field calculated on the pelvis phantom (top row), the prostate SBRT plan calculated on the pelvis phantom (middle row), and the RANDO head and neck IMRT plan (bottom row). CBCT, cone‐beam computed tomography; FFF, flattening filter‐free; IMRT, intensity modulated radiation therapy; SBRT, stereotactic body radiation therapy.

The dosimetric analysis presented in this work relies on the transfer of the CBCT dose distribution to the CT_sim_ dataset, which is accomplished through the CT_sim_‐CBCT image registration. Therefore, any misplacements in the dose transfer due to minor inaccuracies in the image registration may result in larger deviations of *D*_98%_ and *D*_2%_. To account for uncertainties associated with the image registration, a distance‐to‐agreement (DTA) metric was introduced in addition to direct dose difference analysis through three‐dimensional gamma analyses between the CT_sim_‐ and CBCT‐calculated dose distributions. Gamma analysis was performed for the three cases evaluated for a variety of DTA's and dose differences, ranging from 3 to 0.5 mm. All analyses utilize a low‐dose threshold of 5% for which points were considered. A summary of the gamma pass rates as a function of DTA and dose difference is contained in Table 3. For all cases evaluated, the gamma pass rates of ≥90% were observed down to the 1%/1 mm criterion. Figure 7 contains the dose difference and 1%/1 mm gamma analysis maps for the 10 × 10 cm^2^ 6 MV FFF and prostate SBRT fields calculated on the pelvis phantom, in addition to the RANDO head and neck IMRT case evaluated.

**TABLE 3:** Summary of three‐dimensional (3D) gamma pass rates between the CT_sim_‐ and CBCT‐calculated dose distributions for a range of distance to agreement (DTA) and dose difference levels.

Three‐dimensional gamma pass rates (%)					
		Dose difference (%)			
10 × 10 cm^2^, 6 MV FFF field		3	2	1	0.5
DTA (mm)	3	100.0%	99.8%	99.0%	96.8%
	2	100.0%	99.8%	98.5%	95.1%
	1	99.2%	98.4%	94.9%	88.1%
	0.5	96.5%	93.3%	86.5%	77.7%
Prostate SBRT field		Dose difference (%)			
		3	2	1	0.5
DTA (mm)	3	100.0%	99.7%	98.7%	97.7%
	2	99.9%	99.5%	98.0%	95.9%
	1	99.8%	99.1%	95.6%	89.0%
	0.5	99.5%	98.1%	89.1%	71.8%
RANDO head & neck IMRT		Dose difference (%)			
		3	2	1	0.5
DTA (mm)	3	99.9%	99.8%	99.3%	98.5%
	2	99.8%	99.4%	98.2%	96.2%
	1	98.8%	97.1%	90.0%	78.6%
	0.5	94.9%	87.6%	67.5%	46.6%

*Note*: A dose threshold of 5% was used to compute the gamma pass rate.

Abbreviations: CBCT, cone‐beam computed tomography; DTA, distance‐to‐agreement; FFF, flattening filter‐free; IMRT, intensity modulated radiation therapy; SBRT, stereotactic body radiation therapy.

### 3.3 Delivery verification

The subsequent delivery and measurement of the CBCT‐optimized plan demonstrates an end‐to‐end test of HyperSight CBCT‐based treatment planning and optimization. The MU duty cycle on the CT_sim_‐ and CBCT‐optimized plans were 4.66 and 4.81 (total MU/dose per fraction), respectively. Table 4 contains the ionization chamber measurements for both plans across all three arcs. In general, the total dose for both plans was verified to within 0.5% of the calculated dose. For the CT_sim_‐optimized plan, larger inter‐arc variations with respect to measurement were observed on the order of ±6%. For the CBCT‐optimized plan, these inter‐arc variations were reduced, with a maximum difference of 1.8%.

**TABLE 4:** Ionization chamber measurement results at isocenter in a 30 × 30 × 30 cm^3^ stack of SolidWater for CT_sim_ and HyperSight CBCT‐optimized RANDO head & neck IMRT treatment plans.

Plan	Arc #	Measured dose (cGy)	Calculated dose (cGy)	% difference
CT_sim_‐optimized	1	105.3	112.8	−6.7
	2	98.1	92.5	6.0
	3	75.2	74.7	0.6
	Total	278.5	280	−0.5
HyperSight CBCT‐optimized	1	92.3	91.6	0.7
	2	76.0	75.7	0.5
	3	110.7	112.8	−1.8
	Total	279	280	−0.4

Abbreviations: CBCT, cone‐beam computed tomography; IMRT, intensity modulated radiation therapy.

Table 5 contains the EPID and Delta4+ measurement results in terms of gamma pass rate for each arc and the composite measurement at 2%/2 and 1%/1 mm. In general, slightly improved results were observed for the HyperSight CBCT‐optimized plan. Regardless, both plans passed conventional IMRT QA criteria, which, for our clinic, is a pass rate of ≥90% at 3%/2 mm criteria.

**TABLE 5:** Summary of gamma pass rates obtained with EPID‐ and Delta4‐based IMRT QA for the CT_sim_‐ and CBCT‐optimized RANDO head and neck IMRT plans

		Gamma pass criterion and pass rates			
		2%/2 mm		1%/1 mm	
Plan	Arc #	EPID (%)	Delta4+ (%)	EPID (%)	Delta4+ (%)
CT‐optimized	1	99.8	96.0	85.3	75.7
	2	99.9	97.7	94.0	81.2
	3	100	99.0	93.0	78.4
	Composite	100	91.2	91.6	67.5
HyperSight CBCT‐optimized	1	99.8	96.2	93.8	72.9
	2	100	97.1	94.0	79.3
	3	99.9	97.8	92.1	77.6
	Composite	100	93.1	91.9	71.0

*Note*: Pass rates are tabulated at the 2%/2 and 1%/1 mm criterion.

Abbreviations: CBCT, cone‐beam computed tomography; EPID, electronic portal imaging device; IMRT, intensity modulated radiation therapy

## 4 DISCUSSION

This study aims to provide an institutional experience with commissioning and verifying of HyperSight CBCT‐dose calculations—from initial HU to density mapping to comparisons with CT_sim_ calculations through a set of phantom studies. The HU to mass density relationship observed on the HyperSight CBCT was similar to the two conventional fan‐beam CT datasets evaluated, but tended to deviate for non‐water equivalent materials, where maximal differences of ∼150 HU were observed between the SOMATOM go.Open Pro CT simulator and HyperSight CBCT for the same materials. For the Siemens SOMATOM Confidence, deviations in HU were within 100 HU for all materials evaluated. For materials that were near‐water equivalent (1–1.2 g/cc), differences in HU were within 30 HU of each other. Additionally, the influence of iMAR on the derived HU to density curves was minimal, where maximum HU deviations for materials of the same density were < 10 HU for the 125 and 140 kVp scans for all materials evaluated. The largest deviations were shown in the HE Inner Bone insert. It is expected that the influence of iMAR has a negligible impact on the calculated dose distributions, based on results from Davis et al., who showed that HU tolerances of ±20 HU for soft tissue and ±50 HU for lung and bone result in dose changes of < 1% to the treatment plan.^14^ The influence of kVp leads to larger changes in the derived HU to mass density curve for higher density materials. The authors recommend that, for utmost accuracy, all calculations and re‐planning CBCT scans should be acquired using the kVp that was used to measure the HU to density curve. At our institution, CBCTs acquired with the intention of re‐planning are acquired at 125 kVp with the utilization of iMAR if necessary.

Additionally, the emergence of adaptive therapy necessitates an increased emphasis on the monitoring of HU constancy for CBCT imaging systems that may be utilized for adaptive radiotherapy. Although there are no formal recommendations on the frequency with which HU values should be evaluated, we have been performing monthly HU evaluations using the CatPhan 604 (The Phantom Laboratory, Inc., Greenwich, NY). Over the last two years, maximum deviations in HU relative to the two‐year average HU were observed to be up to 13.6. 6.9, 8.9, and 3.8 HU for the Teflon, bone 50%, acrylic, and polymethyl pentene (PMP) CatPhan 604 inserts, which have electron densities (relative to water) of 1.868, 1.312, 1.147, and 0.853, respectively. These HU deviations fall well within the bounds reported by Davis et al. (±20 HU for soft tissue, ±50 HU for lung and bone) to yield dose differences of < 1%.^14^

Dose calculations accuracy of HyperSight CBCT was evaluated against CT_sim_ dose calculations, where the CBCT‐calculated dose was transferred to the CT_sim_ data to evaluate changes in DVH metrics, such as the mean dose and higher‐ and lower‐dose metrics such as *D*_98%_ and *D*_2%_. Through this, it was found that deviations in the mean dose were well within 1% for each of the contours evaluated, while deviations in *D*_98%_ and *D*_2%_ were greater at ∼3% at most. It is hypothesized that elevated deviations *D*_98%_ and *D*_2%_ metrics are mostly due to limitations associated with the image registration and the subsequent CBCT dose transfer to the CT_sim_ image. In other words, *D*_98%_ and *D*_2%_ within the evaluated contours are highly dependent on the registration between the image datasets. Once at distance‐to‐agreement (DTA) metric was introduced through three‐dimensional gamma analysis, all plans and phantoms yielded passing rates of ≥90% down to 1%/1 mm criterion, with a 5% threshold applied.

The results conveyed in this phantom study demonstrate an idealized patient scenario of no anatomical change between CT_sim_ and CBCT imaging, representing a “best‐case‐scenario” clinically. When evaluating dose clinically from a CBCT, the registration with the CT_sim_ image will allow for isocenter placement and structure set transfer between the CT_sim_ and CBCT images. Following the dose calculation on the CBCT, DVH metrics are often evaluated against the CT_sim_ plan. As shown in this work, high‐ and low‐dose DVH metrics, such as *D*_98%_ and *D*_2%_, may exhibit larger deviations (up to ∼3%) while the mean doses were well within 1% of CT_sim_. Therefore, if evaluating dose clinically, changes in the mean dose of greater than 1%, or changes in *D*_98%_ and *D*_2%_ of greater than 3%, would be a strong indication of clinically significant anatomical change. If a gamma analysis workflow is followed for evaluating patient dosimetric change, pass rates of < 90% at 1%/1 mm with a 5% threshold would also indicate potential anatomical change.

When comparing the RANDO head and neck results to those of the pelvis phantom, it is suspected that the reduction in gamma pass rate with RANDO was due to the large number of tissue‐air interfaces introduced with the RANDO phantom, such as those in the oral cavity and esophagus. In addition to these regions of local gamma failure, the slice design of the RANDO phantom can lead to small air gaps between slices, and depending on the imaging dataset used, these slices can affect the local HU and representation of the slice gaps due to volume averaging during the image acquisition, leading to increased discrepancies between the two calculations. In addition to benchmarking HyperSight CBCT against CT_sim_ calculations, end‐to‐end testing of the RANDO head and neck IMRT plan demonstrates the feasibility and accuracy of HyperSight CBCT‐based planning.

The results conveyed in this study indicate that daily CBCT imaging on the HyperSight platform can be used to gauge treatment progression through a patient's course of treatment through offline analysis of doses calculated on daily CBCT scans. Future work will focus on leveraging automated CBCT dose calculations to progress towards a dose accumulation workflow.

## 5 CONCLUSIONS

This study provides a comprehensive overview of the dosimetric accuracy of Varian's HyperSight CBCT platform available on the Halcyon treatment machine at our institution. Through this work, it was found that HyperSight dose calculations are accurate to within 1% of CT_sim_. Our goal is that this institutional experience will serve as a benchmark for others in developing online and offline CBCT‐guided radiation therapy programs using HyperSight CBCT.

## AUTHOR CONTRIBUTIONS

The author contributions to this article are as follows: study design and conception: N. Nelson and H. Zhao; data collection: N. Nelson, H. Zhao, and C. Oare; data analysis and interpretation: N. Nelson, H. Zhao, C. Oare, G. Nelson, T. Martin, J. Huang; manuscript drafting and preparation: N. Nelson, H. Zhao, C. Oare, G. Nelson, T. Martin, J. Huang.

## CONFLICT OF INTEREST STATEMENT

The authors declare no conflicts of interest.

## Data Availability

The data that support the findings of this study are available from the corresponding author upon reasonable request.
